# Fabrication of Nanostructured Cadmium Selenide Thin Films for Optoelectronics Applications

**DOI:** 10.3389/fchem.2021.661723

**Published:** 2021-04-07

**Authors:** Shahnwaz Hussain, Mazhar Iqbal, Ayaz Arif Khan, Muhammad Nasir Khan, Ghazanfar Mehboob, Sohaib Ajmal, J. M. Ashfaq, Gohar Mehboob, M. Shafiq Ahmed, Said Nasir Khisro, Chang-Jiu Li, Raphael Chikwenze, Sabastine Ezugwu

**Affiliations:** ^1^State Key Laboratory for Mechanical Behavior of Materials, School of Materials Science and Engineering, Xi'an Jiaotong University, Xi'an, China; ^2^Department of Physics, University of Azad Jammu and Kashmir, Muzaffarabad, Pakistan; ^3^Central Diagnostic Laboratory (CDL), Physics Division, Pakistan Institute of Nuclear Science and Technology (PINSTECH), Islamabad, Pakistan; ^4^Department of Physics, University of Kotli Azad Jammu and Kashmir, Kotli, Pakistan; ^5^School of Materials Science and Engineering, South China University of Technology, Guangzhou, China; ^6^Department of Physics, Faculty of Sciences, Alex Ekwueme Federal University Ndufu-Alike Ikwo, Abakaliki, Nigeria; ^7^Department of Physics & Astronomy, University of Western Ontario, London, ON, Canada

**Keywords:** CdSe, thin films, chemical bath deposition, optical properties, energy materials, electrochemical energy storage

## Abstract

There is lot of research work at enhancing the performance of energy conversion and energy storage devices such as solar cells, supercapacitors, and batteries. In this regard, the low bandgap and a high absorption coefficient of CdSe thin films in the visible region, as well as, the low electrical resistivity make them ideal for the next generation of chalcogenide-based photovoltaic and electrochemical energy storage devices. Here, we present the properties of CdSe thin films synthesized at temperatures (below 100°C using readily available precursors) that are reproducible, efficient and economical. The samples were characterized using XRD, FTIR, RBS, UV-vis spectroscopy. Annealed samples showed crystalline cubic structure along (111) preferential direction with the grain size of the nanostructures increasing from 2.23 to 4.13 nm with increasing annealing temperatures. The optical properties of the samples indicate a small shift in the bandgap energy, from 2.20 to 2.12 eV with a decreasing deposition temperature. The band gap is suitably located in the visible solar energy region, which make these CdSe thin films ideal for solar energy harvesting. It also has potential to be used in electrochemical energy storage applications.

## Introduction

Cadmium Selenide is an n-type direct bandgap II-VI semiconducting material. The bulk bandgap energy of 1.74 eV at 300 K is very close to the NIR, which can be increased through a variety of processes (Acharya et al., 2010). The reported molecular weight is 191.37 g/mole in which Cd is 58.74% and Se is 41.26% and has a dark red color in appearance (Acharya et al., 2010). During the last decade, extensive research work has been carried out on the II-VI group semiconductors such as CdSe, CdTe, ZnSe, and CdS thin films due to their potential applications in optoelectronic devices. CdSe is an important member of this group of compounds because of its extensive use in different fields such as biomedical technology, solar cell technology (Pal et al., 1990; Gruszecki and Holmström, 1993), chemical sensing, thin film transistors (Hossain et al., 2019), photoconductors (Shimizu et al., 1971), acousto-optical devices (Bonello and Fernandez, 1993), gas sensors (Smyntyna et al., 1994), photoelectrochemical devices (Naushad et al., 2018), and photoreceptors. Different fabrication techniques have been reported to grow thin films of CdSe which includes the chemical bath deposition technique (Erat et al., 2008; Hernandez-Perez et al., 2008), molecule beam epitaxy (Samarth et al., 1989), electrodeposition (Sturgis, 2015), spray pyrolysis, successive ionic layer adsorption and reaction (Pathan et al., 2003), thermal evaporation (Baban and Rusu, 2003; Shreekanthan et al., 2003), and MOVCD (Chae et al., 2006).

Extensive research work has been done on the structural, electrical, and optical properties of CdSe thin films, but it still demands further research for a variety of reasons. Although, semiconducting CdSe can be utilized for application in solar cells (Rickus, 1982) electrochemical energy storage devices (Joonho Bae and Dong Kee, 2016), and other optoelectronic devices (Fan et al., 2015) their competitiveness and use in such devices demand a fabrication method which is both reliable, cost-effective, and scalable. Many laboratory-based researches abound but very little has been done to transition from basic or fundamental research to develop reliable prototype devices and possible commercialization (Zhao et al., 2012). Most times, these basic researches are conducted with materials and apparatuses that are cost-intensive and the condition of use of such appliances are outrageously high, like very high temperatures (Chan et al., 2005), ultra-high vacuum (Bernard et al., 2004), etc. These extreme fabrication conditions can be a big challenge when transitioning from laboratory to industry. Therefore, there is an urgent need for reliable techniques that will allow for easy control of different parameters or the deposition conditions and to tune and optimize the properties of CdSe thin films to make them more efficient for utilization and application in devices.

The chemical bath deposition technique (CBD) (Chikwenze and Ezugwu, 2015; Ike et al., 2019) is considered as the most suitable technique for the fabrication of semiconducting CdSe thin films owing to many advantages such as cost-effective, homogeneous films, simple, large area deposition and its ability to get good thin films using parameters that can be easily controlled.

The influence of bath temperature on structural, morphological, chemical composition and optical properties of CdSe thin films was investigated by Vishwakarma et al. (2013). Thickness dependent variation in structural, optical and electrical properties of CdSe thin films was also studied (Choudhary and Chauhan, 2019). Effect of deposition potential on the morphological and optical properties was studied (Bai et al., 2020). Crystal structure, surface profile and optical properties of CdSe thin films deposited at different bath temperatures was also investigated (Li et al., 2018). But the effect of bath temperatures also effect of annealing on structural, morphological and optical properties has not been investigated.

In this paper, a low-temperature fabrication of nanostructured thin films of CdSe via simple, cost and time effective CBD method is reported. Special attention has been paid to the preparation of CdSe thin films by keeping the temperature below 100°C. After successful preparation of CdSe thin films, their structural, interfacial, and optical properties have been studied. Effect of substrate temperatures and annealing temperatures on the optoelectronic and structural properties of these films are explored. It has been found that the CdSe thin films absorb visible light (within the wavelength range of 563–585 nm), which corresponds to the bandgap energy of 2.12–2.20 eV. While a high vacuum sputtered CdSe thin film exhibited NIR bandgap energy, our low-cost fabrication method yields films that can harvest visible light energy for photovoltaic application. Furthermore, the SEM study has revealed particles agglomeration, and increase in particle size from 230 to 680 nm with an increasing deposition temperature which is in conformity with the temperature dependence of the particle size evolution report in the literature (Rosly et al., 2021). This ability to deposit CdSe semiconductor thin films at low temperatures below the glass transition temperatures of many flexible substrates has the potential to open new perspectives for studying, in a reliable manner the electronic and optical property of semiconducting thin films on these substrates.

## Experimental Details

### Sample Preparation

CdSe thin films can be prepared by using several precursors of Cd and Se. In this work we used cadmium acetate dehydrate [Cd(CH_3_COO)_2_.2H_2_O] (99% purity), selenium powder (Se) (99.9% purity), Triethanolamine (TEA) [N(CH_2_CH_2_OH)_3_] (98% purity, sodium sulphite (Na_2_SO_3_) (98% purity) and 25% ammonium hydroxide (NH_4_OH) as the precursors. Substrate cleaning is vital in the fabrication of thin films to enhance the adhesion of the films. The glass slide substrates were first washed with detergent and water, kept in hydrochloric acid (a suitable amount of HCL is taken in which commercially available glass slides were immersed vertically) for 2 h, washed in deionized water and then rinsed in acetone. Ultrasonic cleaning in deionized water for 15 min was subsequently carried out as the last step in our cleaning process. Well-cleaned glass substrates were placed in a hot oven for a few minutes to dry.

**Figure 1:**
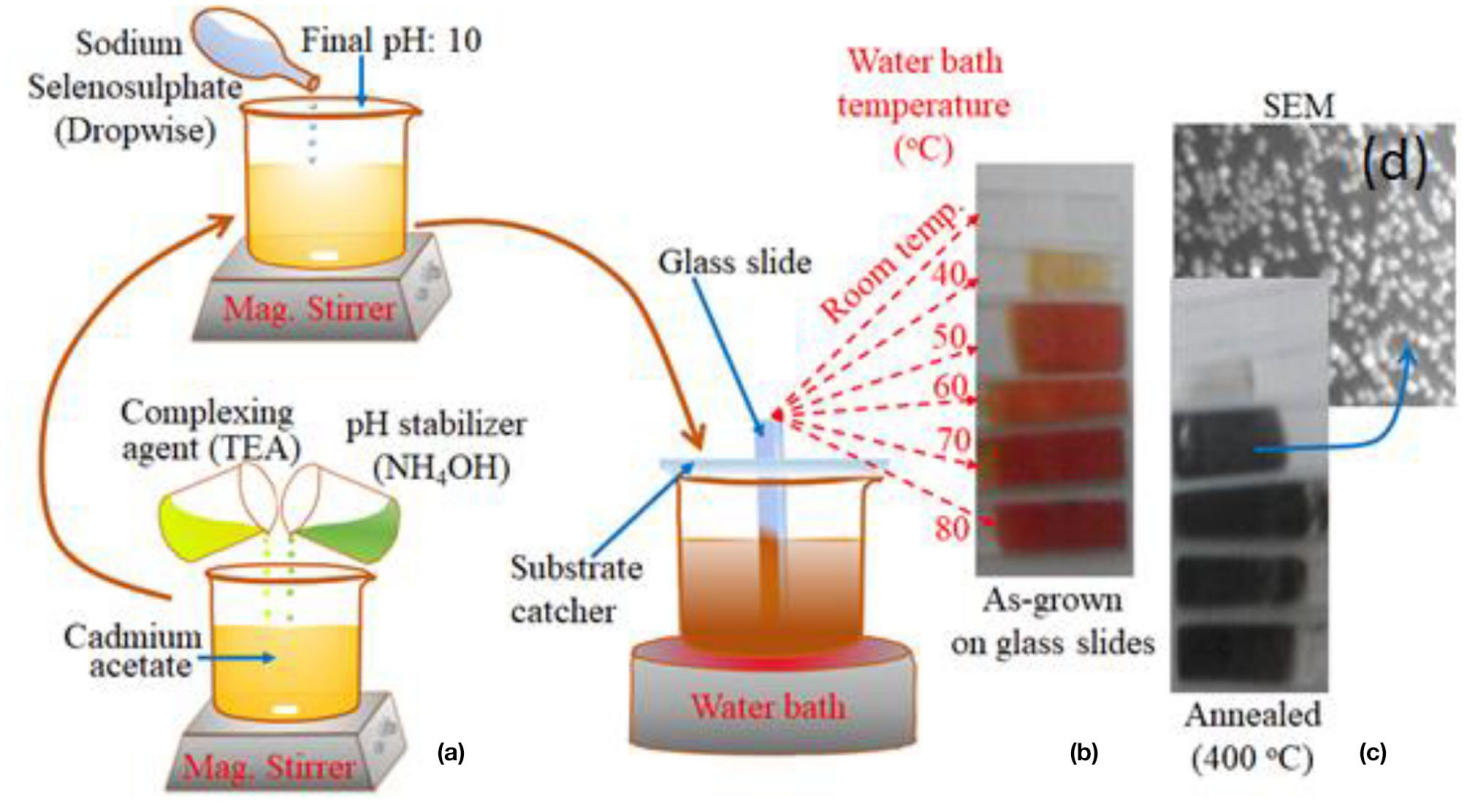
**(a)** The stepwise procedure for the fabrication of CdSe thin films by chemical bath deposition method using the complexing agent, pH stabilizer and freshly prepared Se salt. **(b)** Picture of five samples of CdSe thin film deposited on the glass substrate at different deposition bath temperatures. **(c)** Picture of the samples of CdSe annealed at 400°C, and **(d)** SEM image of CdSe sample.

Prior to the deposition of CdSe thin films, aqueous solution of the various precursors was prepared. Cadmium acetate dihydrate [Cd(CH_3_COO)_2_.2H_2_O] serves as the source of Cd ions while freshly prepared sodium selenosulfate (Na_2_S_e_SO_3_) was used as the source of Se ions. For preparing its solution, 1.89 g of sodium sulphite (Na_2_SO_3_) was dissolved in 15 ml of water. Then 1.84 g of selenium (Se) was added to this solution. The solution was kept at the temperature of 85°C for 5 h with constant magnetic stirring. A fresh solution of sodium selenosulfate is required because the salt is unstable if allowed to stand for a long time in solution. Figure 1a shows the stages involved in the growth of CdSe thin films in which 0.5 mol.L^−1^ of cadmium acetate dihydrate was first dissolved in a beaker with 10 mL of water. The solution was subsequently placed in a magnetic stirrer set at a low stirring speed. Five ml of triethanolamine (TEA) which serves as the complexing agent was introduced to the solution dropwise while stirring. To stabilize the pH of the solution, 10 ml of 25% aqueous ammonia was added into the beaker, followed by thorough mixing with the magnetic stirrer. Next, 15 ml of freshly prepared sodium selenosulfate solution was added, dropwise, with the pH of the solution kept constant at 10 ± 0.5.

A homemade substrate catcher was used to place the substrate centrally in the solution as shown in Figure 1a. Thin films of CdSe was deposited on the glass substrate for 6 h at fixed temperature, which was maintained by a water bath apparatus. This procedure was repeated to grow other samples at different temperatures of 40, 50, 60, 70, and 80^o^C, respectively. A new precursor solution was used for each tested temperature. A control sample at room temperature was also deposited as shown in Figure 1b. After the deposition, the slide was washed with a fountain of deionized water to eliminate residues before drying them in air. It is observed that films of CdSe which are prepared at room temperature were colorless but as the bath temperature increased colorless CdSe thin film changed gradually to orange and then to dark red as shown in Figure 1b.

To obtain thin films with good crystallinity, the samples were annealed in a tube furnace at temperatures of 100, 200, and 400°C for 2 h. It was observed that after annealing, the color of the CdSe films turned to dark black consistently with previous reports (Ali et al., 2013). The set of samples that were annealed at 400°C are depicted in Figure 1c. Typical SEM image of annealed samples shown in Figure 1d indicates that the thin films comprised of well-nucleated nanoparticles.

### Characterization

The structural and optical characteristics of these films were investigated as a function of the annealing temperatures. Structural and phase analysis of CdSe thin films were done by using Rigaku Geiger flux instrument with Cu K_α_ radiations having wavelength 1.54051 Å. The XRD patterns of these films were obtained in angular range (2θ) from 10° to 80° with a step size of 0.05°. FTIR transmissions spectra of CdSe thin films synthesized at different bath temperatures and annealed at 400°C were obtained with Perkin-Elmer FTIR Spectrometer. The spectra were taken at room temperature in the range of 400–4,000 cm^−1^. The Surface morphology of the thin films was examined with a scanning electron microscope (Jeol: JSM-6510LV), which was operated at 25 kV. The optical properties were investigated with Perkin Elmer Lambda 19 Spectrometer operated at the wavelength range from 300 to 800 nm.

The chemical composition of CdSe thin films and interface structure for interdiffusion between CdSe films and glass substrate were studied using Rutherford Backscattering Spectroscopy (RBS). The RBS was carried out by utilizing a 5 MeV pelletron tandem accelerator. The accelerator generated a mono energetic He^++^ ions beam, which has energy 2.084 MeV with a beam current of 28 nA. The RBS data simulation was carried out by using ion beam analysis software (SIMNRA).

## Results and Discussion

### Formation of CdSe

The deposition of CdSe thin film using CBD technique involves controlled precipitation of the ions in solution and subsequent nucleation of the resulting species on the substrates such as the microscopic glass slides used in this study. At optimum reaction temperature, the precipitation of CdSe occurs when the ionic product of reactants becomes greater than their solubility product. However, if the conditions for precipitation are not fully satisfied the resulting solid phase dissolves in the solution and ultimately resulting in a depleted or no deposition (Ezugwu et al., 2009; Deshpande et al., 2013a; Onyia, 2017).

The pH of the solution, the deposition temperatures and the complexing agent concentration are the key parameters that control the concentration of the free metal ions in CBD setups. By controlling the release of Cd^2+^ and Se^2−^ ions, one can obtained uniform thin films at optimum deposition conditions. In this work, triethanolamine (TEA) was used as the complexing agent to regulate the hydrolysis of cadmium metal ion in the solution. TEA was added dropwise to the cadmium salt to initiate a slow reaction in the solution and subsequent formation of complex Cd salt as shown below:

$$\begin{array}{l} \begin{array}{cc} Cd^{2+}+TEA\to {\left[Cd\left(TEA\right)\right]}^{2+} & \; \end{array} \end{array}$$

Ammonia was dissolved in water to provide OH^−^ ions which forms part of the reacting species:

$$\begin{array}{l} NH_{3\;}+H_{2}O⇌NH_{4}^{+}+OH^{-} \end{array}$$

The overall chemical reaction for the deposition of thin films of CdSe can be described by the following chemical equation.

$$\begin{array}{l} {\left[Cd\left(TEA\right)\right]}^{2+}+Na_{2}SeSO_{3}+2OH^{-}\to CdSe+Na_{2}SO_{4}\\ +TEA+H_{2}O \end{array}$$

To enhance the deposition rate and tune the properties of the resulting thin films of CdSe, the deposition was carried out at different temperatures by placing the solution bath on a regulated water bath. Six samples of films were fabricated on glass substrates at different temperatures of 40, 50, 60, 70, 80°C and a control sample at room temperature.

### Structural Studies

**Figure 2:**
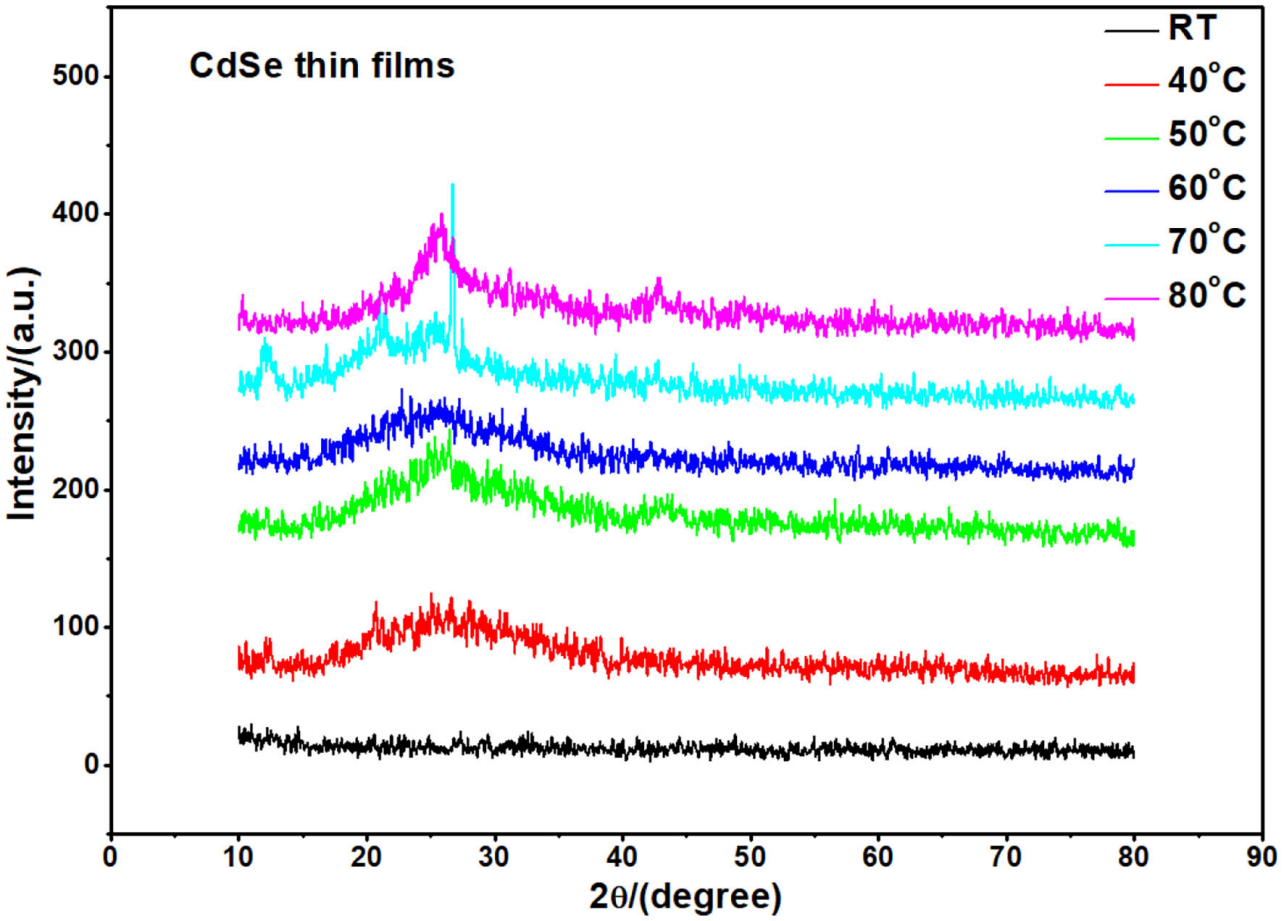
XRD pattern of as-deposited thin films of CdSe fabricated at different bath temperatures. The samples appear amorphous in structure.

**Figure 3:**
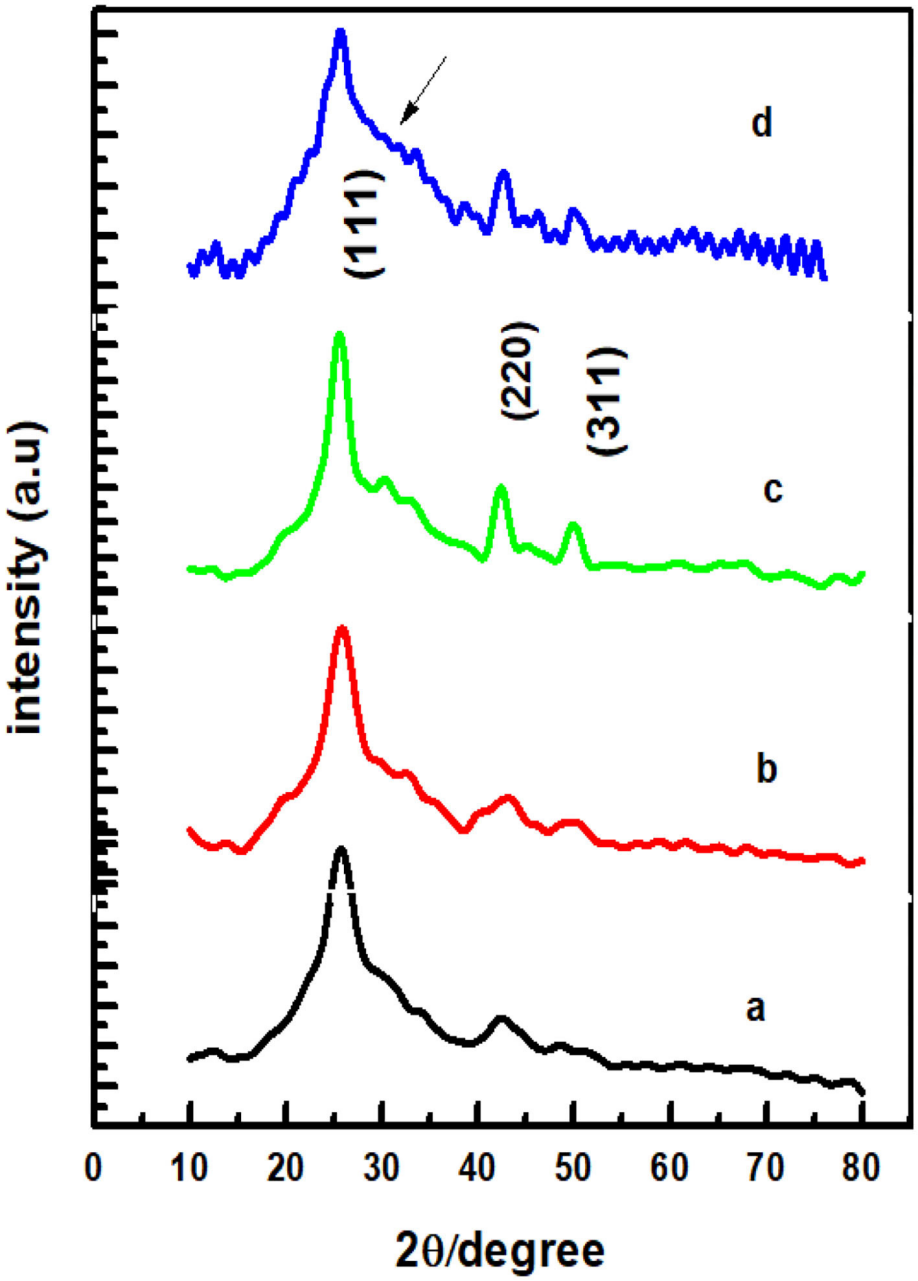
XRD patterns of CdSe thin films deposited at bath temperature of 80°C and annealed in air at **(a)** 100°C, **(b)** 200°C, **(c)** 400°C, and **(d)** 450°C.

CdSe has two structural polymorphs, the crystal structure appearing as Wurtzite or cubic, zinc blende-type (Xia et al., 2010). A previous report (Kale and Lu, 2015) show that the thin films of CdSe deposited on a glass substrate by CBD is either cubic or hexagonal. In our specific case, the XRD pattern of the films deposited at different bath temperatures shown in Figure 2 revealed that the samples are mostly amorphous in nature. However, as the deposition temperature increases, a small-broadened peak starts to appear. The broad XRD peak around 25.5°C is clearly observed for the thin films synthesized at 80°C. It appears that the best bath temperatures for the thin film deposition are 70 and 80°C. Hence, the sample deposited at 80°C will be investigated further. It was observed that the thermal treatment of the samples helps to induce structural modification from amorphous to crystalline, as shown in Figure 3. It is clear from these results that the CdSe films deposited at 80°C and annealed at 100, 200, and 400°C are polycrystalline in nature and have a cubic structure with intense (111) reflection peaks. Figure 3 also shows that as annealing temperature increase from 100 to 400°C the crystallinity also increases. However, the sample which was annealed at 450°C showed an increased broadening of the (111) diffraction peak. This is possibly due to a decrease in the crystallite size at this elevated annealing temperature (see Table 1). When CdSe thin films are annealed at a temperature of 450°C, chemical degradation can occur with a possible conversion from CdSe to the oxide, CdO (Mahato and Kar, 2017).

**Table 1:** The parameter of the CdSe structure calculated from the XRD results.

**Materials with annealed temperatures**	**2θ (degree)**	**(hkl)**	**Lattice constant (a) (Å)**	**FWHM (β) (radian)**	**Grain size (D) (nm)**	**Dislocation density (δ) × 10^**16**^ (lines/m^**2**^)**	**Micro strain (ε) × 10^**−3**^**
CdSe (100°C)	25.588	111	6.1297	0.0638	2.23	20.13	15.55
CdSe (200°C)	25.588	111	6.2094	0.0540	2.63	14.41	13.15
CdSe (400°C)	25.340	111	6.0102	0.0343	4.13	5.84	8.38
CdSe (450°C)	25.596	111	6.0208	0.2115	0.67	21.25	51.56

The crystalline size of the nanocrystallites was calculated by using Scherrer's formula given below.

$$\begin{array}{l} D=\frac{k\lambda }{\beta \cos\theta } \end{array}$$

where “*D*” is crystallite size, *k* is constant having value 0.9, λ is the wavelength of X-rays, *β* is full width at half maximum, and *θ* is Bragg's angle. Dislocation density (δ) and microstrain (ε) of CdSe thin films were also calculated with the following equations:

$$\begin{array}{l} \delta =\frac{1}{D^{2}}\\ \varepsilon =\frac{\beta \cos\theta }{4} \end{array}$$

The values of the Lattice constant (a), d spacing, FWHM (β), grain size (D), dislocation density (δ), and microstrain (ε) calculated from the XRD results in Figure 3 are reported in Table 1. The results show that as the annealing temperatures of thin films of CdSe increases the full width at half maximum, FWHM (β) of the films decreases. Consequently, the grain size of the samples increases from 2.23 to 4.13 nm with annealing temperatures. However, the dislocation density (δ) and microstrain (ε) of the films decreases with the increase in the annealing temperature. The intensity of the peaks also increases by increasing the annealing temperature.

### FTIR Studies

**Figure 4:**
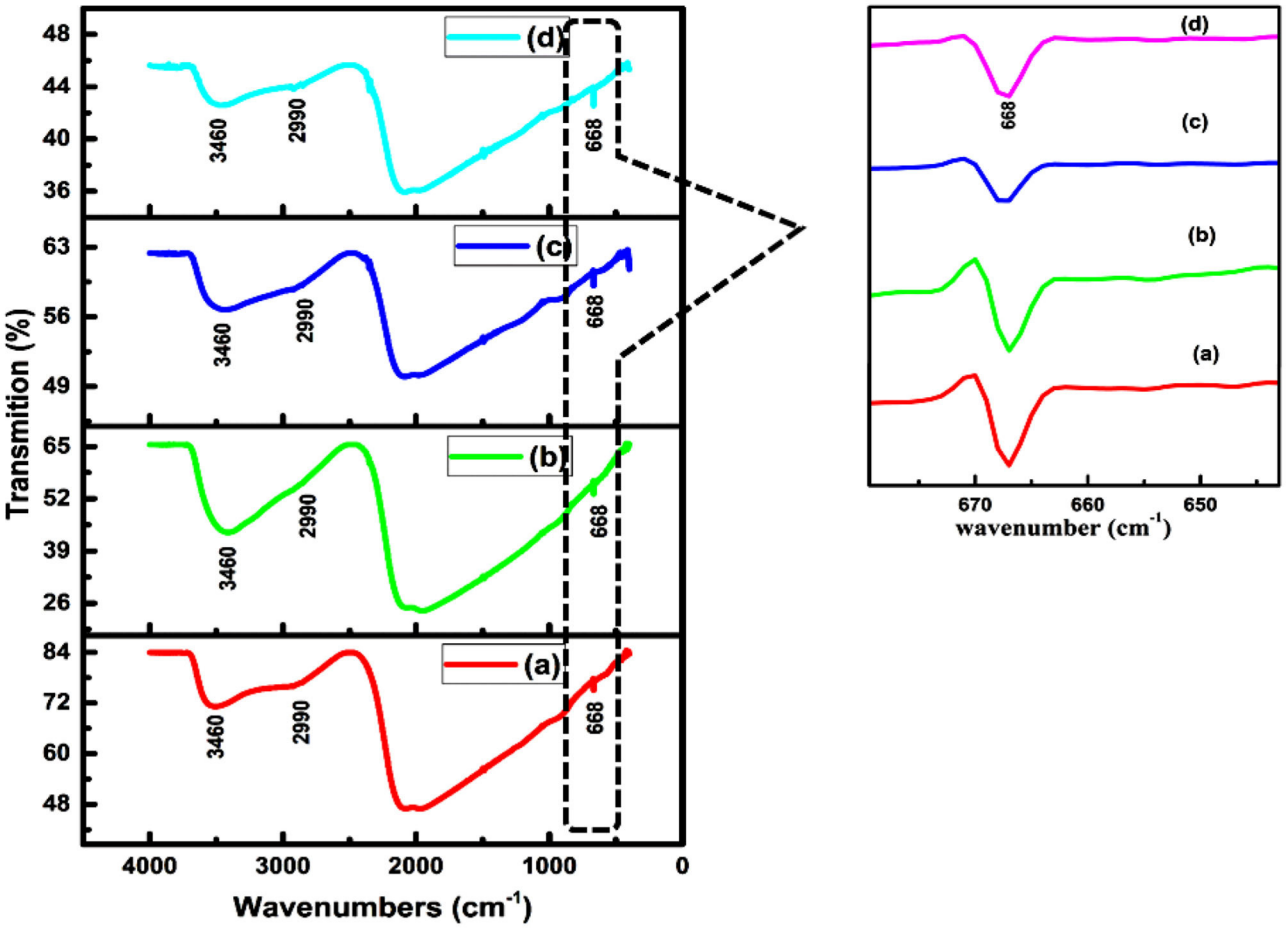
FTIR pattern of CdSe thin films deposited at **(a)** 50°C, **(b)** 70°C, **(c)** 80°C, and **(d)** annealed at 400°C.

Fourier transform spectroscopy is used to investigate vibrational modes of different finctional groups. Figure 4 shows the FTIR pattern of thin films of CdSe deposited at bath temperatures of (a) 50°C, (b) 70°C, (c) 80°C, and (d) film synthesized at 80^o^C and then annealed at 400°C. The broad peak observed at 3,460 cm^−1^ is assigned to O-H stretching and is due to the presence of a small quantity of water molecules in CdSe thin films. The peak at 2,990 cm^−1^ is assigned to C-H stretching of CH_2_ group (Ravindranadh et al., 2013). The peak observed at 668 cm^−1^ is the fingerprint spectral peak of CdSe (Rani, 2014). This band confirmed the occurrence of a vibrational mode of CdSe lying between 400 and 700 cm^−1^ (Ali et al., 2013). Figure 4 also revealed that the peak which is due to O-H stretching shows decreasing %T intensities by increasing the deposition temperatures from 50 to 80°C and by annealing at 400°C.

### Surface Morphology

**Figure 5:**
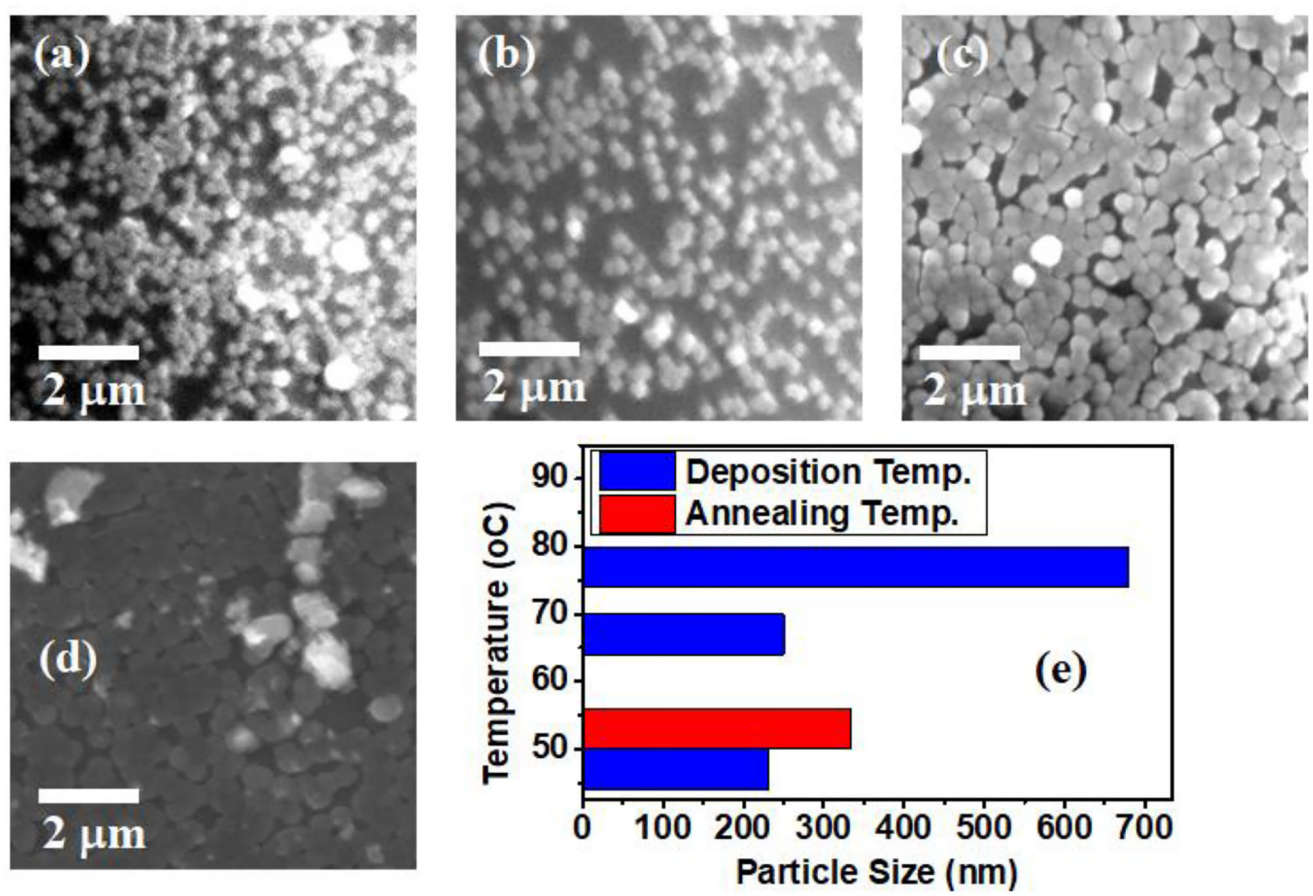
SEM micrographs of CdSe thin films synthesized at different bath temperatures of **(a)** 50°C, **(b)** 70°C, **(c)** 80°C, **(d)** CdSe deposited at 50°C, and annealed at 400°C. **(e)** Histogram of particles size distribution as a function of deposition and annealing temperatures.

Scanning electron microscope (SEM) which provides an appropriate way to study the surface morphology of thin films was used to examine the surface morphology of our samples. SEM gives information about grain size, roughness, and surface morphology of thin films. Figures 5a–c show SEM micrographs and surface morphology of thin films of CdSe synthesized at different bath temperatures of 50, 70, and 80°C. From the SEM micrograph of these films, it is evident that films adhered well to the surface of the substrate and consist of many small grains that are sparsely distributed. However, the density of the nanoparticles appears to increase with the increasing temperature of the deposition bath. Figure 5d shows that post-deposition thermal annealing has significant effects on the morphology of our CdSe thin films. A comparison of Figures 5a,d indicates that the morphology of the sample annealed at 400°C is dense and compact. This can be attributed to the increase in the activation energy needed for intergrain diffusion, leading to smaller grains joining to form large grains, which in turn increases the size of the nanoparticles as shown in Figure 5e.

Figure 5e shows that the particle size also depends on the deposition temperature, increasing from 230 nm at 50°C deposition temperature to as much as 680 nm at 80°C. We observe a significant increase of the size of nanoparticles for the samples deposited at 80°C when compared to the two samples deposited at lower bath temperatures of 50 and 70°C. This observation suggests that 80°C is the ideal bath temperature in which nucleation and growth of Cd and Se ions on the substrates can proceed favorably to form nanoparticles. Once the initial nuclei are attached on the substrate, further growth from the impinging ions in the condensation is expected to proceed via the Volmer-Weber growth pattern, leading to the observed increase in both the density and size of the nanoparticles (Apeh et al., 2019).

### Optical Transmission Analysis of Thin Films of CdSe

**Figure 6:**
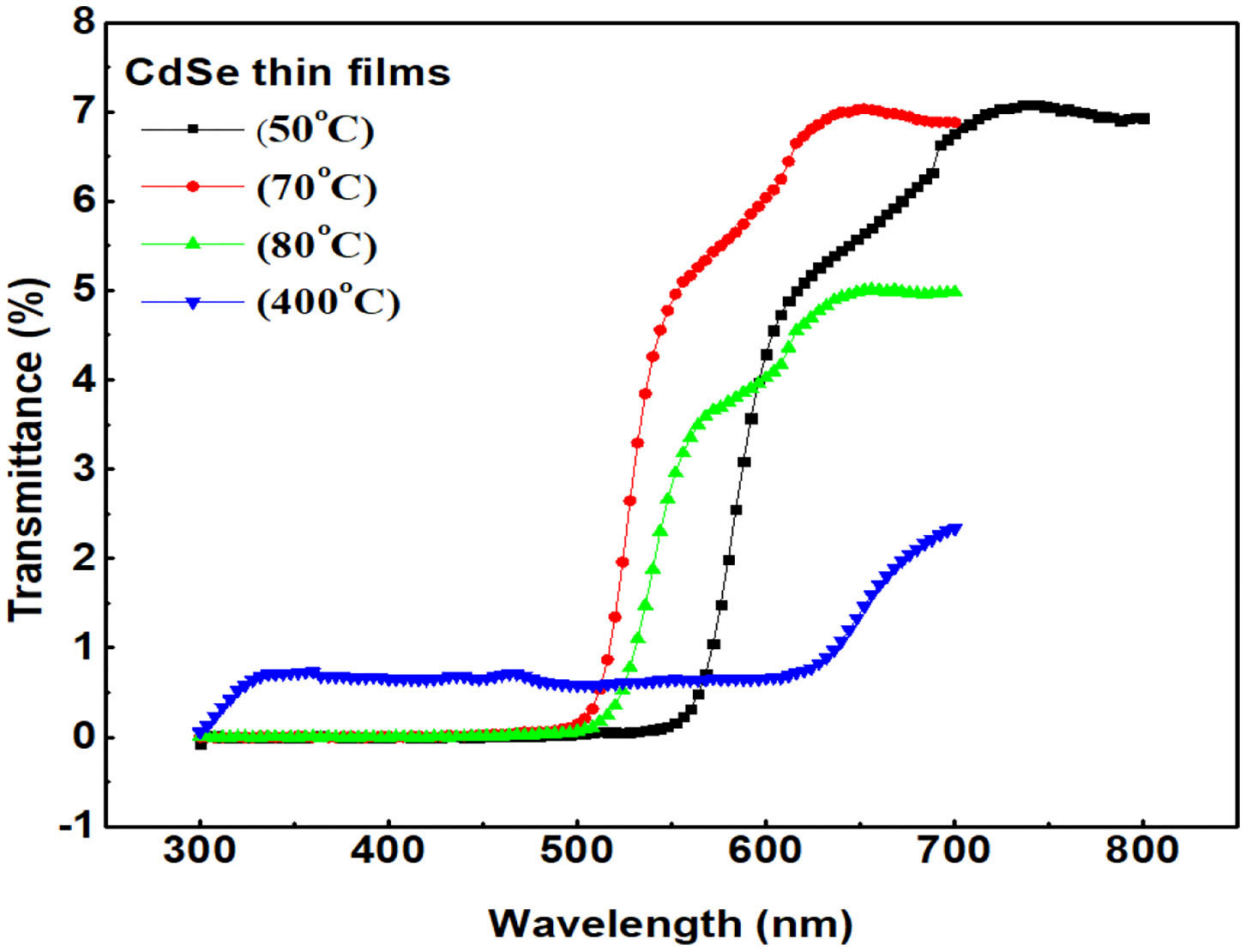
Transmittance of thin films of CdSe synthesized at 50, 70, 80, and annealed at 400°C.

Ultraviole-tvisible (UV-vis) spectroscopy is an optical technique used to investigate optical aborption/transmission of the sample. Figure 6 represent transmittance spectra of CdSe thin films deposited at different bath temperatures and annealed at 400°C. The figure indicates that the transmittance of thin films of CdSe is generally low, approaching 10 % at maximum. The figure shows that the transmission spectra of CdSe thin films have relatively higher transmittance (>5%) above 500 nm (in the visible region), and near-infrared region, 700–800 nm. In UV region, 350–400 nm, the transmittance of CdSe thin films decrease to a very low value (<2%), indicative of very high absorbance in the UV region of electromagnetic radiation. The result of our UV-Vis measurements shows that the transmittance of thin films of CdSe depends on the deposition and annealing temperatures. From the graphs shown in Figure 6, the transmittance decreases as deposition temperature increases, consistently with our earlier observation that the density and compactness of the samples increase with bath temperatures. The decrease in the transmittance of CdSe samples with increasing bath temperature is also consistent with the observed color changes of the films in Figure 1, from light red to dark red, indicative of greater interdiffusion of isolated grains and particle growth increase with increasing bath temperature. The transmittance of the annealed sample is also very low as compared to the un-annealed samples. After annealing, the dark red color of the thin film of CdSe changes to dark black as shown previously in Figure 1. Besides the color changes, our SEM analysis shows that post-deposition thermal annealing increases the diffusion energy for the grains to form large particles, which leads to denser films (Jamil et al., 2012). The observed color changes from dark red to dark black, the particle growth and increased density are responsible for the huge decrease in the transmittance of our CdSe thin films.

### Optical Band Gap Energy of CdSe Thin Films

**Figure 7:**
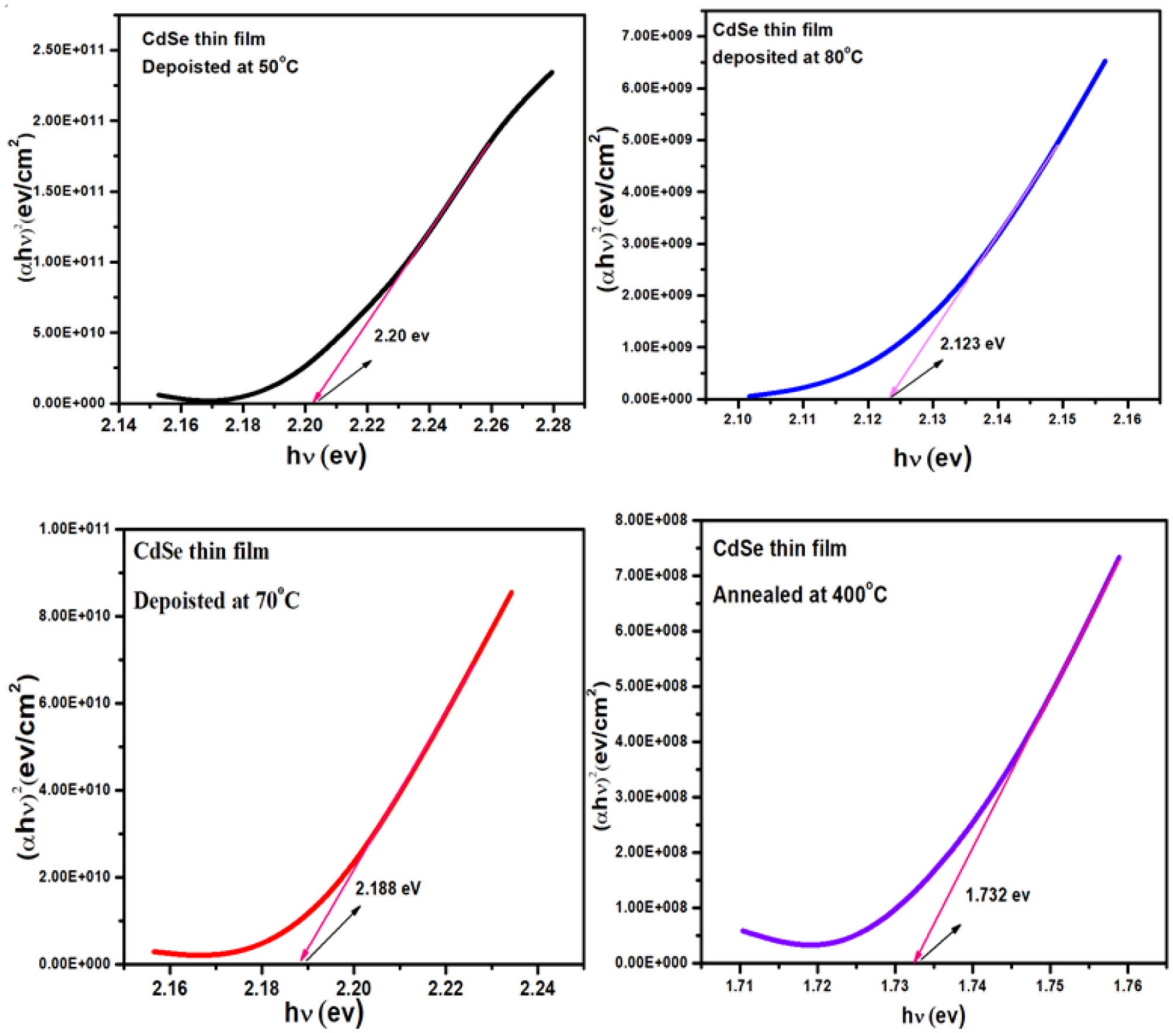
Plot of (αhν)^2^ vs. (hν) of thin film of CdSe deposited at different temperatures and the film deposited at 80°C and annealed at 400°C.

Figure 7 represent the graphs of (αhν)^2^ vs. *h*ν for the thin film of CdSe deposited at different bath temperatures of 50, 70, and 80°C and later annealed at 400°C. The energy bandgap of the CdSe films was determined by extrapolating the straight-line part of the plot to zero absorption making an intercept with the energy axis. The interception on the energy axis (hν) gives the value of the energy bandgap. Figure 7 shows that the graphs of the bandgap energy are linear over a wide range of energy, which confirms that CdSe thin films deposited in this work, are direct bandgap semiconductor (Bernard et al., 2004). Values of the energy band gap of as-synthesized thin films of CdSe at different temperatures decrease from 2.20 to 2.12 eV as illustrated in Table 2. El-Menyawy and Azab reported similar values for CdSe thin films prepared by the thermal evaporation technique (El-Menyawy and Azab, 2018). In our case, the bandgap energy tends to decrease with high temperature thermal annealing. In fact, after annealing at 400°C values of the energy band gap of CdSe thin film decreased further to 1.73 eV in agreement with the bulk CdSe and consistently with the observed low value of the optical transmittance with thermal annealing. The decrease in the energy band gap can be attributed to the quantum confinement in CdSe thin films due to the increase in crystallite size and a decrease in defects in these films (Deshpande et al., 2013b). Visual inspection shows that the films exhibited color changes upon thermal annealing from dark red to gloomy black in conformity to earlier work reported in the literature (Daniel et al., 2017).

**Table 2:** Optical parameters of thin films of CdSe calculated from the optical spectra.

**Material with deposition temperature**	**Band gap value of bulk CdSe (E**_****G****_**)** **=** **1.74 eV,** ** Wavelength (λ** **=** **712.6 nm)**				
	**Film thickness (nm)**	**Wavelength (λ) (nm)**	**Band gap (E) (eV)**	**ΔEg = E-E_**G**_ (eV)**	**Grain size (nm)**
50°C	100	563.63	2.20	0.46	2.396
70°C	120	566.72	2.188	0.44	2.488
80°C	280	588.079	2.123	0.38	2.626

The average diameter of nanocrystallites can be calculated from the blue shift of band gap of CdSe, which was examined in the samples prepared at different bath temperatures, by using the following equation (Mehta et al., 2007).

$$\begin{array}{l} \text{Δ}E_{g}=E_{g}\left(film\right)-E_{g}\left(bulk\right)=E_{shift}=\frac{\hbar^{2}\pi^{2}}{2\mu R^{2}} \end{array}$$

where μ is the translation mass and is equal to (m_h+_ m_e_), E_shift_ is bandgap shift and R is the radius of the crystallites. By putting the observed values of band gaps of CdSe thin films from the band gap value of bulk CdSe and putting the standard values of other parameters in the above equation, the diameter of the crystallites of CdSe can be determined. The crystallite (grain) size of CdSe, which were deposited at a temperature of 50, 70, and 80°C are found to be 2.396, 2.488, and 2.626 nm, respectively. These values are close to the grain sizes determined from the XRD diffractogram reported previously in Table 1.

Values of the bandgap, wavelength, band energy shift, the crystallite size and some of the other optical parameters of CdSe thin films calculated from the optical spectra are shown in Table 2. We observe that the thickness of CdSe thin films increases with increasing deposition temperature. The grain size of the films also increases and consequently the energy band gap decreases as the deposition temperature increases. These results show that the increase in the crystallinity of the films agrees with the SEM micrographs reported previously in Figure 5. The bandgap energies of the samples deposited at different temperatures are situated in the visible light of the solar spectrum. As such, the thin films are suitable for optoelectronic applications, especially in a solar cell where they can be used to generate photocurrent from visible light absorption.

### Rutherford Back Scattering Spectroscopy

**Figure 8:**
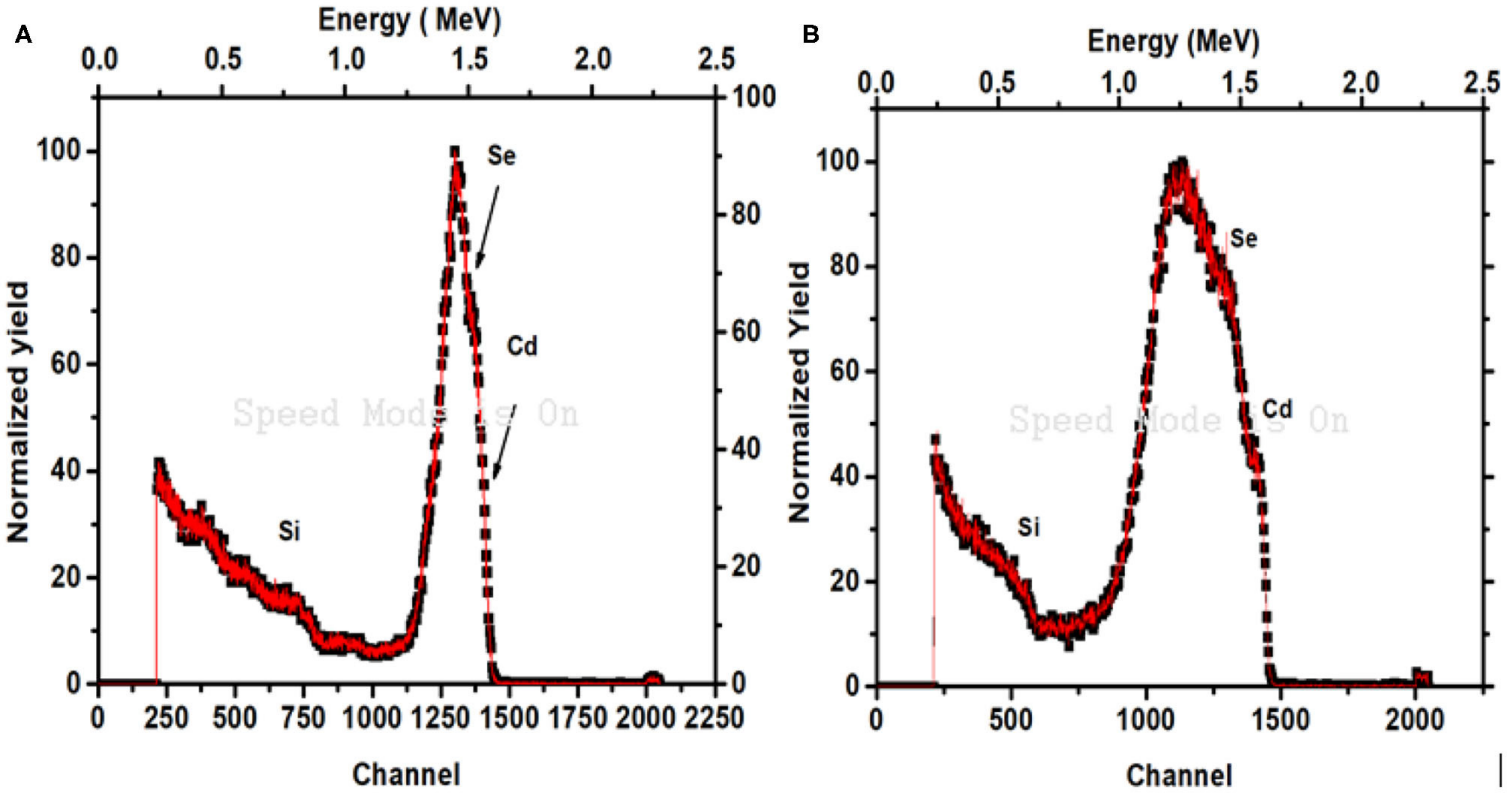
RBS spectra of CdSe thin films deposited at **(A)** 70°C, **(B)** 80°C.

Rutherford Back Scattering Spectroscopy (RBS) is useful technique that is used to investigate the elemental profile of Cd and Se along the thickness of the prepared thin films. Figure 8 shows the RBS spectra of thin films of CdSe which were deposited at 70 and 80°C, respectively. Apart from the Si signal arising from the glass substrate, two other peaks of Cadmium (Cd) and selenium (Se) are emanating from the thin films. From the RBS graph, it is seen that resolution of observed peaks for Cd and Se is very poor which is due to interdiffusion between CdSe thin films and glass substrate of these few nm thick films. As a result, the composition analysis of our sample could not be calculated with the help of RBS spectra due to the poor resolution of the Cd and Se peaks. However, the film thickness was calculated by the model fitting of the RBS data using software SIMNRA and has been reported in Table 2.

## Conclusions

CdSe films with few nanometer thicknesses were successfully deposited by chemical bath deposition technique on the glass substrates at low temperature (<100°C). The XRD results show that CdSe thin films deposited at different bath temperatures were initially amorphous in nature but after annealing at elevated temperatures, they transformed to polycrystalline thin films having cubic structure. The crystallite size increases from 2.23 to 4.13 nm as annealing temperatures increase from 100 to 400°C. FTIR spectroscopy confirmed the peak of CdSe at 668 cm^−1^. The RBS results showed two peaks of Cd and Se but the exact composition could not be extracted due to the huge overlap of the peaks. SEM images of the films revealed spherical nanoparticles. By increasing the deposition and annealing temperatures, small grains agglomerate together to form larger grains, which significantly influenced the optical properties of the films. The results of UV-Vis spectroscopy of CdSe thin films showed that the transmittance decreases with increasing deposition and annealing temperatures. By increasing the deposition temperatures, the bandgap values decreased from 2.20 to 2.12 eV and after annealing at the temperature of 400°C the bandgap energy further decreased to 1.73 eV. The values of the bandgap energy of the sample deposited at low temperature, below 100°C is located in the visible region of the solar spectrum. Hence, the films can be used in solar energy architecture as energy harvester.

## Data Availability Statement

The original contributions presented in the study are included in the article/supplementary material, further inquiries can be directed to the corresponding author/s.

## Author Contributions

All authors listed have made a substantial, direct and intellectual contribution to the work, and approved it for publication.

## Conflict of Interest

The authors declare that the research was conducted in the absence of any commercial or financial relationships that could be construed as a potential conflict of interest.
